# SARS‐CoV‐2 Omicron variant: A next phase of the COVID‐19 pandemic and a call to arms for system sciences and precision medicine

**DOI:** 10.1002/mco2.119

**Published:** 2022-02-11

**Authors:** Ebrahim Mostafavi, Ankit Kumar Dubey, Laura Teodori, Seeram Ramakrishna, Ajeet Kaushik

**Affiliations:** ^1^ Stanford Cardiovascular Institute Stanford University School of Medicine Stanford California USA; ^2^ Department of Medicine Stanford University School of Medicine Stanford California USA; ^3^ Institute of Scholars Chikmagalur Karnataka India; ^4^ Diagnostics and Metrology Laboratory FSN‐TECFIS‐DIM, ENEA Frascati Roma Italy; ^5^ Center for Nanotechnology and Sustainability National University of Singapore Singapore; ^6^ NanoBioTech Laboratory, Health Systems Engineering, Department of Natural Sciences Florida Polytechnic University Lakeland Florida USA

**Keywords:** COVID‐19 infection, diagnostics, nanomedicine, Omicron, precision medicine, SARS‐CoV‐2 mutations

## Abstract

Since early 2020, coronavirus diseases 2019 (COVID‐19) infection pandemic/endemic is constantly surprising health experts because of continuous variations in the structures of severe acute respiratory coronavirus 2 (SARS‐CoV‐2) in the form of newly emerged variants. Such mutations have exhibited high mortality and severity due to the newly emerged more infectious sites of SARS‐CoV‐2, making viral infection more transmissible, infectious, and severe. Recently, SARS‐CoV‐2 mutated to another variant, namely, Omicron (B.1.1.529), which is many times more transmissible and infectious than existed deadly Delta variants of the virus. This severity is closely correlated to a larger number of mutations observed in the receptor‐binding domain of the spike protein of the Omicron‐SARS‐CoV‐2. Considering severity, Omicron has been declared as variant of concerns by the World Health Organization and within days from its emergence, Omicron infection has spread globally, increased hospitalization, exhibited more severity for the young generation, invaded defense mechanism of natural immunity, not responsive to the available vaccines. Such circumstances resonated with the efficiency of available strategies established to manage COVID‐19 intelligently and successfully. To explore these aspects, this perspective article carefully and critically summarizes the Omicron's origin, structure, pathogenesis, impact health along with health systems, and experts’ recommendations to manage it successfully.

## Graphical abstract

In this perspective, the authors first discussed the Omicron's origin, mutations, structure, pathogenesis, and its impact on human health while comparing it with the previous variant of concerns (VOCs). System science and precision medicine are introduced by employing Artificial Intelligence (AI), big data reservoirs, bioinformatic systems, advanced in vitro 3D models, and subtypes sciences to diagnose and eradicate the Omicron virus. Experts’ recommendations to successfully manage Omicron‐SARS‐CoV‐2 variant are also provided.

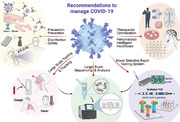

## 1 INTRODUCTION

**FIGURE 1:**
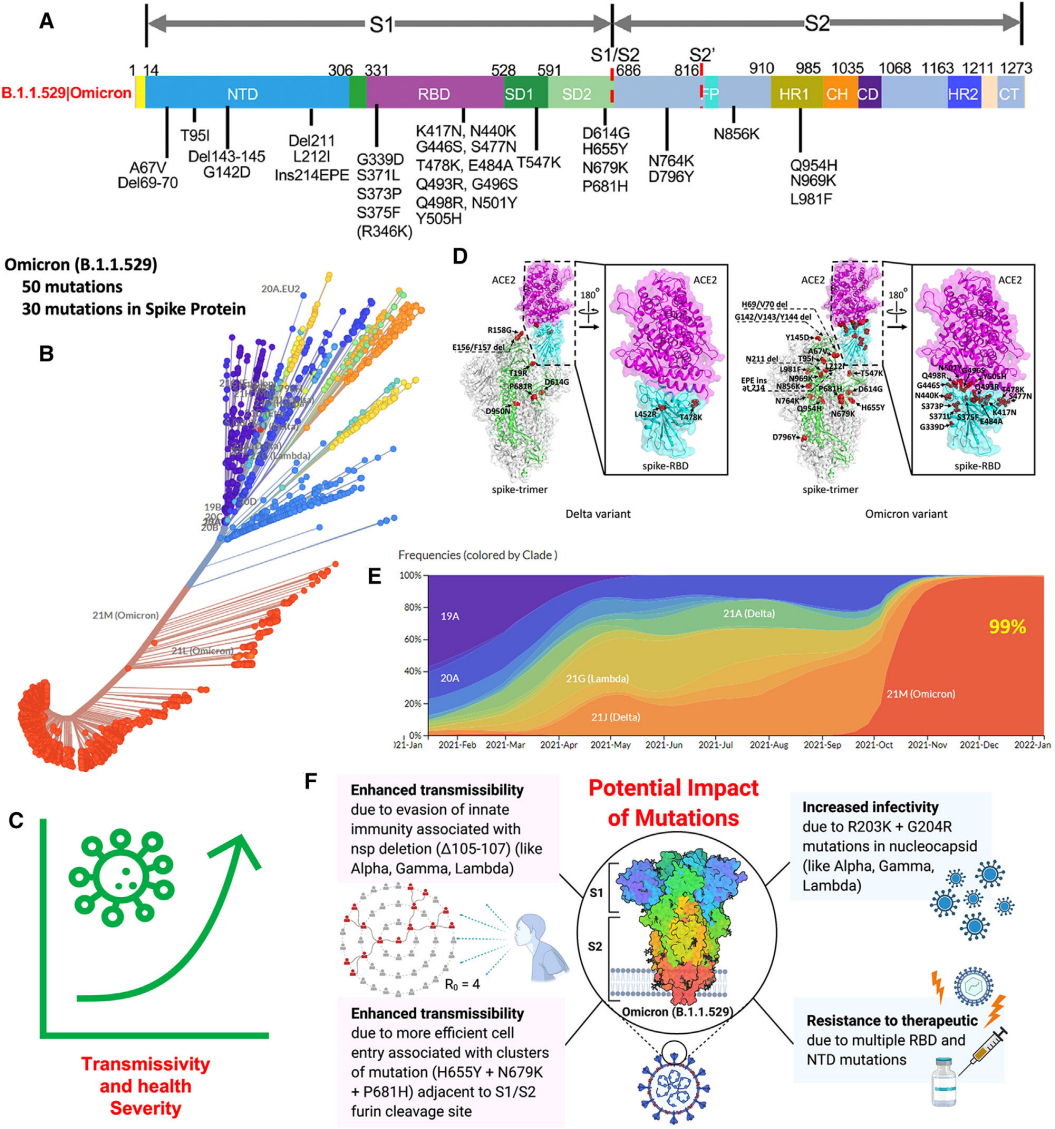
Illustration of Omicron mutation and its effects on health, infection progression, and COVID‐19 management aspects. (A) The main spike mutations found from the isolated viruses of Omicron B.1.1.529, Copyright permission Nature 2022.^4^ (B) Phylogenetic analysis of SARS‐CoV‐2 clusters mutation (*Source*: https://nextstrain.org/groups/neherlab/ncov/21K.Omicron?l = radial). (C) Total 50 mutations in which 30 of them at spike protein causing transmissivity and severe health effect. (D) Illustration of spike protein mutation of Omicron (right side) in comparison of Delta (left side), Copyright permission Wiley 2022.^13^ (E) Frequency of infection over the time, since December 2021 Omicron is contributing 99%, and Delta is around 1% (*Source*: https://nextstrain.org/groups/neherlab/ncov/21K.Omicron?l = radial). (F) The potential impact of Omicron (B.1.1.529) causing enhanced transmission, infectivity, and resistance to therapeutics, especially vaccines. Figure F is created using BioRender.com

New SARS‐CoV‐2 variant, namely, Omicron (B.1.1.529), was first reported by the Network for Genomics Surveillance in South Africa on November 24, 2021, which had been detected from collected South African samples from November 14, 2021.^1^ Only 2 days after its report (on November 26, 2021), Omicron was declared as variant of concern by the World Health Organization (WHO) technical advisory group, and it diverted experts debate about whether the COVID‐19 pandemic is under control or a more infectious phase is yet to begin. Among Omicron's 50 new mutations, 30 investigated at Spike (S) proteins (Figure 1A) have surprised epidemiologists, virologists, and infectious disease experts because such mutated variant is expected to be more transmissible,^2^ infectious, and potent to invade state‐of‐art therapeutics strategies, including vaccines^3^, ^4^, ^5^, ^6^, ^7^ (Figure 1B,C). By January 18, 2022, Omicron variants are confirmed in more than 145 nations, and the infection spreading rate is getting double within 2–3 days, for example, in the United Kingdom, where Omicron has been declared as an emergency and considered a serious concern to Europe due to its potency of broader spreading. Presently, the health agencies, like WHO, NIH, CDC, and so on, are being focused on discussing the seriousness of Omicron variants and its comparison with the severity of Delta variants (which was the reason for the second deadliest wave of COVID‐19 pandemic).^8^

**The severity of Omicron variants infection and its comparison with other variants should be the two separate topics of investigation**. Critical understanding of recent investigations suggested everyone to consider Omicron infection seriously without waiting for more information as the world did during the emergence of Delta variants. To provide better clarity, this commentary explores Omicron variants emergence, health impacts, expected challenges, predictions, and alternative approaches to control associated COVID‐19 infection (proposed COVID‐19 third wave). This can pave the way for how to approach the other upcoming variants.

There is no clear explanation about Omicron's origin except theories like random mutation among several types of COVID‐19 infection, zoonotic mutation involving human‐to‐animal‐to‐human, and association of SARS‐CoV‐2 with seasonal flu virus correlate to the observed surprising gap between Delta and Omicron variants genomic profiling.^9^ Regardless of doubts, these Omicron's alterations will cause severe health consequences to immunocompromised people, including those recovering after COVID‐19 and the fully vaccinated population. In this direction, experts are exploring pathways of Omicron infection to decipher the infection mechanism and variant's ability to escape immunizations and induce reinfections. Such investigations are crucial as 30 alterations at S‐protein can easily detect host cells to affect human inflammatory and immune responses.^10^, ^11^ The unusual mutations (among 30, 26 S‐mutations investigated the first time) of Omicron stud S‐protein receptor‐binding domain (RBD), the part that interacts with human cells before cell entry, make Omicron noticeable concern. These sites are the primary target for vaccines that significantly improve binding interactions in the RBD–hACE2 complex while still having caused rapid spread in large populations.^11^ Omicron's S‐mutations allow it to be discovered by genotyping assays, which produce findings considerably faster than genome sequencing.^9^, ^12^

## 2 STRUCTURE OF SARS‐COV‐2 OMICRON SPIKE PROTEIN

The detailed structural explanation of Omicron variants (substitution/deletions/insertions) is well explained by He et al. along with the differences in comparison to Delta variants as illustrated in Figure  1D.^13^ The study explained that Delta variants are more contagious, but Omicron variant is more transmissible and infectious. However, the less % of severity cannot be the reason to overlook the severe health consequences that originated with Omicron infection because Omicron exhibited 15 mutations accommodated in the RBD (a site responsible for bonding with receptors, i.e., ACE‐2 enzyme to facilitate the virus entry to the host cell), where the Delta has only two such mutations, namely, L453R and T478K (Figure 1D). Omicrons 15 contagious mutation related to virus entry makes its infection and associated pathogenesis more transmissible and infectious. As a result, the viral infection started increasing rapidly, and recently (since January 2022), the frequency of Omicrons infection is more than 90% with reference to the total COVID‐19 infection globally (Figure 1E). Many investigated SARS‐CoV‐2 mutations make the Omicron variant resistive to protective care and treatments, including vaccines. Therefore, Omicron infection exhibited enhancement in infection and transmission, as illustrated in Figure 1F.

**FIGURE 2:**
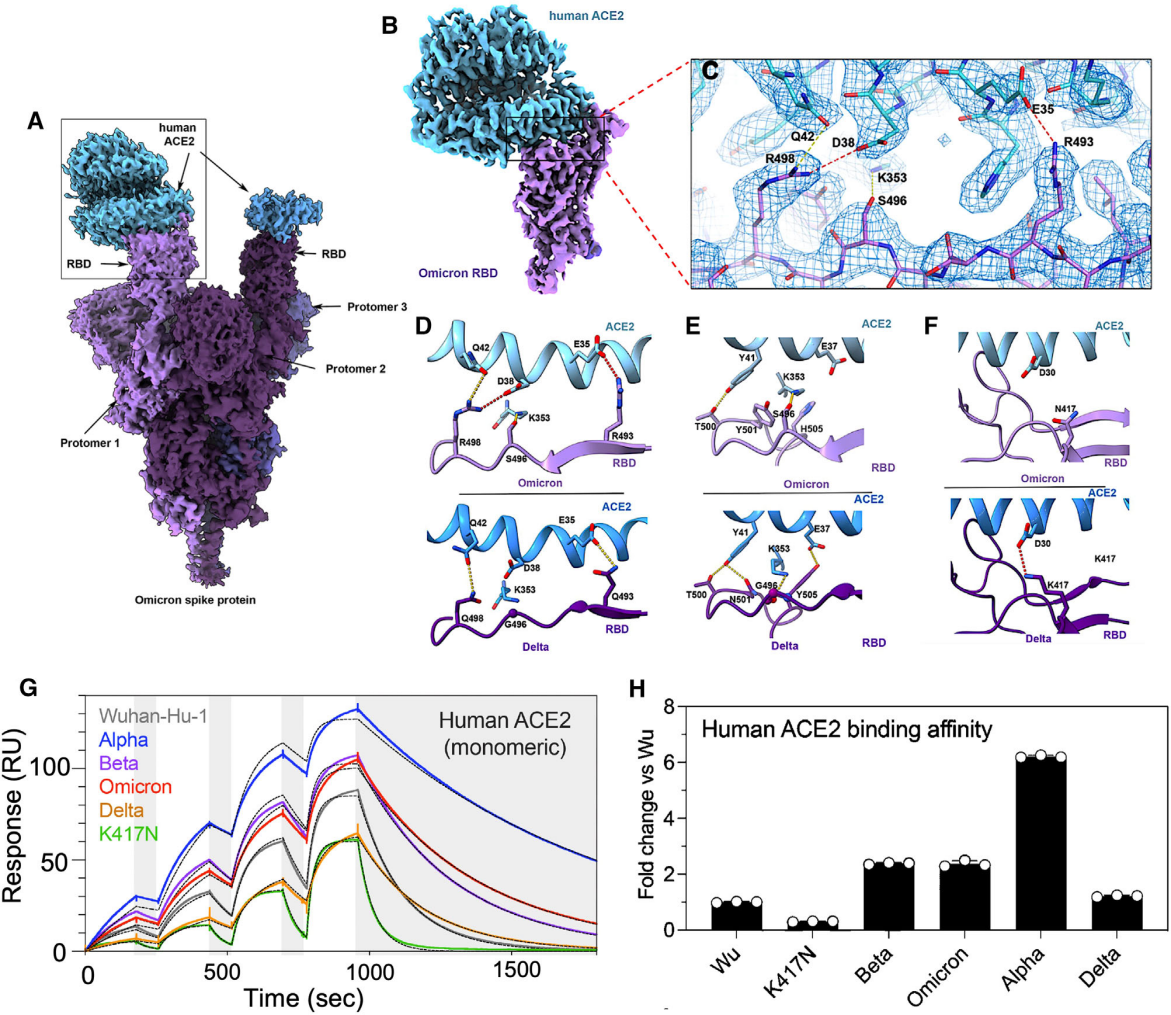
Omicron spike protein–human ACE2 complex interactions. (A) Cryo‐EM structural map of the Omicron spike protein in complex with human ACE2 at 2.45Å resolution. Human ACE2 is colored in blue, while various shades of purple are used for three different promoters. (B) Cryo‐EM structural map of the Omicron spike RBD in complex with ACE2 at 2.66 Å resolution, where (C) shows the fitted atomic model of Cryo‐EM density mesh at the highlighted inset of (B). (D–F) Comparison of the RBD–ACE2 interface between the Omicron (top panels) and Delta (bottom panels) variants reveals that in the case of Omicron variant, new interactions are formed, which is attributed to new mutations, namely, Q493R, G496S, and Q498R, Copyright permission Science 2022.^2^ (G,H) Omicron variant RBD shows increased binding to human ACE2. (G) ACE2 binding to six RBD variants, and comparison between their single‐cycle kinetics surface plasmon resonance (SPR) analysis. (H) Quantification of human ACE2 binding data, Copyright permission Nature 2022^7^

Although the newly reported Omicron variant has a large number of mutations and is currently the most prevalent SARS‐CoV‐2 variant worldwide, a new Cryo‐EM structural analysis of the SARS‐CoV‐2 Omicron spike protein in complex with human ACE2 revealed very interesting results^2^ (Figure 2A–F). The study shows that despite having many mutations, Omicron spike protein binds to human ACE2 with an affinity similar to Delta variant (Figure 2G,H), which arises from a series of interactions that compensate for other Omicron mutations (such as K417N) that are known for reducing ACE2 binding affinity.^2^ The neutralization assay results also showed an increased antibody evasion in the new Omicron variant of SARS‐CoV‐2, which is in accordance with other reports.^3^, ^4^, ^5^, ^6^, ^7^ Of note, for Beta or Delta variants, there was no population‐wide epidemiological evidence showing any kind of immune escape.^14^ Therefore, the increase in antibody evasion, together with maintaining the binding interaction at the ACE2 interface, leads to understanding a particular molecular feature that most likely contributes to Omicron's increased transmissibility.^2^

Another important concern is how effectively Omicron can defy past infection immunity or COVID‐19 vaccinations and create a surge outbreak.^8^ Recent reports claim that Omicron variants can decrease vaccines efficacy because their altered active and blocking sites may confuse the therapeutic mechanism of vaccines.^9^ Therefore, a modification in available vaccines and developing more efficient boosters are urgently required based on large‐scale genomic profiling of Omicron to know exact targeting sites.^15^ Besides, an impairment has been observed in this variant of Spike's fusogenicity, which leads to a lower formation of syncytes (syncytium are agglomerations of multinucleated cells produced by the fusion of two or more cells) and to a reduction in the replication of transfected cells in lung tissues; however, it is not known yet if other organs or systems might be involved.

## 3 OMICRON‐SARS‐COV‐2 MANAGEMENT

Due to Omicron's numerous mutations discussed above, this variant has acquired greater immune evasion than the previous ones, that is, it is partially resistant to both vaccine neutralizing antibodies, monoclonal antibodies, and those derived from natural immunity.^3^ Recent reports also suggest that the concept of natural and vaccine‐induced immunity will work differently in the case of Omicron infection. Therefore, the first thing to properly manage the Omicron variant is the diagnosis of the Omicron patients. As a good point, PCR and antigen COVID‐19 tests are the most reliable diagnostic approaches that are still effective in detecting Omicron variant as it is associated with S‐protein. However, these tests are time‐consuming and expensive; therefore, sincere efforts are required for rapid testing kits specific to Omicron. Proposals are available in the literature that can be tailored on the variant(s) and perhaps ready to be marketed soon. For instance, nanofiber swabs that are shown to reduce false‐negative results compared to classical flocked and cotton swabs. The use of nanoparticles for the isolation of RNA or DNA from biological samples via magnetic field can also be an alternative method for rapid diagnosis. These nanoenable SARS‐CoV‐2 sensors successfully detected virus concentration at a low level (picomolar level) selectively.^16^ Not only the smart nanosystem but numerous efficient biomarkers, such as antibodies, CIRSR/Cas,^17^ and segment selective DNA/RNA, have also been investigated for the selective detection of SARS‐CoV‐2 even in a real sample. However, these nanotechnology‐supported biosensing systems need a lot of validation and population‐based confirmation before they can be recommended for clinical and point‐of‐care (POC) testing.^18^

Besides, these nanobiosensors have not yet been tested against the Omicron, which must be the priority. State‐of‐the‐art SARS‐CoV‐2 biosensors work against the spike protein, so they should work in theory.^19^ Since Omicron has several mutations at spike protein compared to other SARS‐CoV‐2 variants, a well‐designed and high‐throughput validation of available diagnostics tool must be presented for diagnosing a large population and confirming whether COVID‐19 infection due to Omicron or not.

The artificial intelligence (AI) and internet of medical things (IoMT)‐supported biosensing approach has emerged as a top recommendation of health experts to utilize the information of viral load for understanding the diseases progression and treatment efficacy assessment.^20^, ^21^ In the presented unprecedented scenario, AI and IoMT‐supported biosensors to detect Omicron variants can be part of telemedicine to analyze the health consequences of an individual with reference to medical condition/profiling.^20,21^ There connecting all these aspects, the personalized Omicron variants infection progression can be monitored in a personalized manner.

**FIGURE 3:**
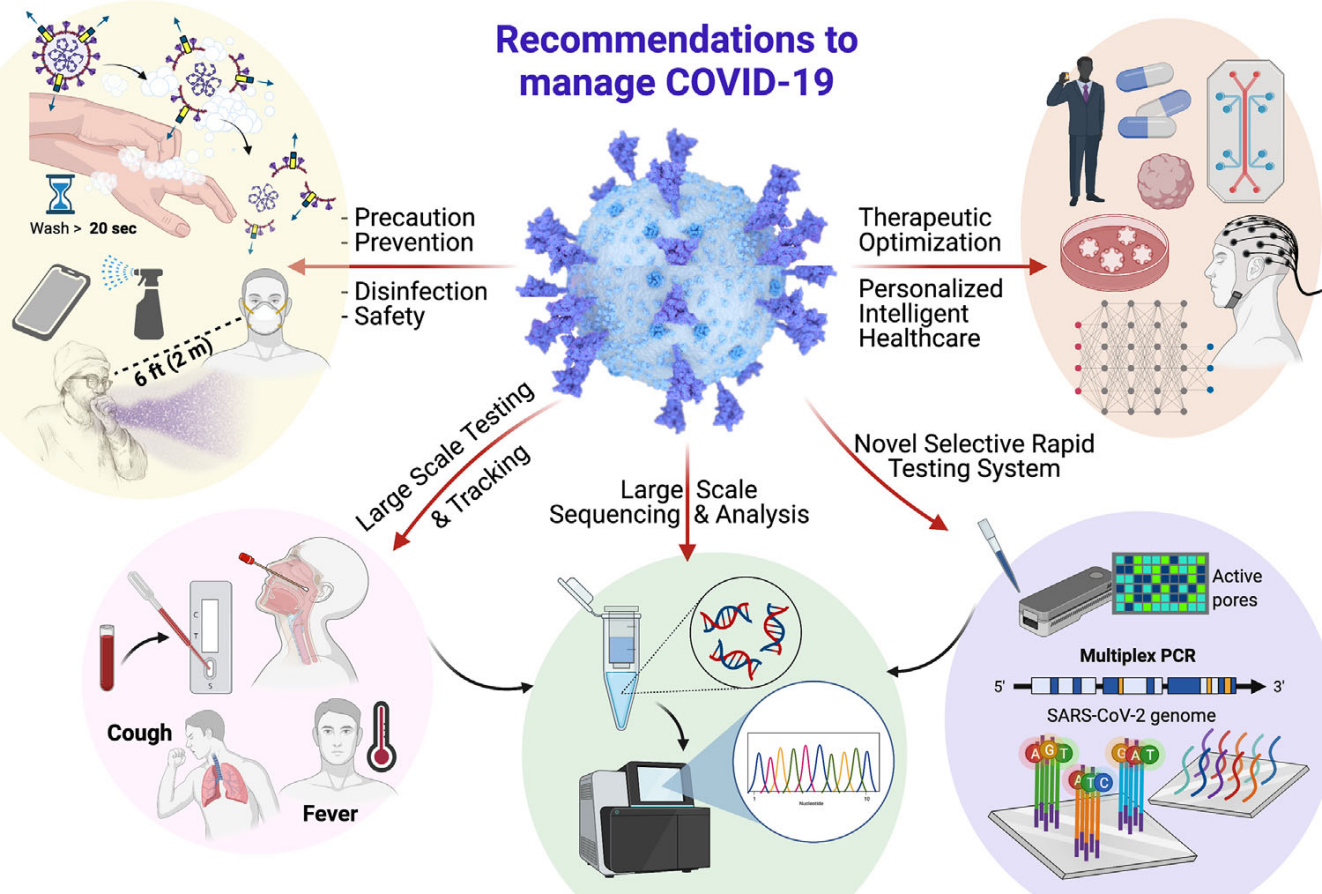
Recommendations to manage Omicron‐SARS‐CoV‐2 variant. Experts recommend managing Omicron infection by taking every precaution seriously, increasing rapid and selective testing of Omicron, tracking of infected and postinfection patients to understand pathogenesis and cross‐reactivity, fostering novel therapeutics to recognize and eradicate Omicron virus, and precision medicine for individuals by employing AI, big data reservoirs, bioinformatic system, advanced in vitro 3D models (microfluidic systems, organoids, etc.), and subtype sciences. Figure created using BioRender.com

Considering health consequence followings, usual precaution hygiene should be followed and protection devices should be used (to avoid air born and hospital acquired transmission), masking and physical distance (human‐to‐human transmission), limited travel (to avoid regional transmission), mass testing (for diagnostics and bioinformatics), and tracking of patients (for safety and diseases progression monitoring) are suggested for safe society, community, and working environment together with all other efforts to maintain alive economic society (Figure 3). Home testing campaigns to reduce virus transmission among the population, although already initiated, should be fostered. Furthermore, the high‐risk population could be monitored with more sophisticated wearable devices that could potentially and significantly reduce the proportion of transmissions by presymptomatic individuals through the use of wearable devices and appropriate algorithm analysis.^22^ On the other hand, a series of interesting multidisciplinary approaches ready to market or bench to bedside or POC platforms could help manage the Omicron variant more appropriately.

Great attention should also be devoted to personalized/precision medicine to develop more effective methods of diagnostics and therapeutics. Due to the dramatic velocity of virus evolutionary changes, multiple predisposing host factors influence disease susceptibility and course and response. A full vaccination followed by the booster dose to manage COVID‐19 infection has proven its potential. As a result, the hospitalization and COVID‐19 infection were reduced drastically because these developed therapies efficiently recognize and eradicate SARS‐CoV‐2. Nanotechnology is an incredible support for optimizing treatments to increase developed vaccines or therapies.^23^, ^24^ As SARS‐CoV‐2 viral pathogenesis affects various organs of the human system, investigating the therapeutics that can tackle SAR‐CoV‐2, infection‐associated side‐effects, and support the postinfection recovery of a patient is an urgent need. To achieve this, there is an unmet need to investigate novel pharmacological relevant therapeutic cargo involving every aspect of nanomedicine. In the case of Omicron, there is an urgent need for engineering available vaccines to combat Omicron‐SARS‐CoV‐2 mutations. In this direction, such engineered vaccines can be delivered at a target site using nanomedicine approaches.^23^, ^25^, ^26^, ^27^ Nanomedicine can also be engineered to manage Omicron along with postinfection consequences with the help of another therapeutic agent, and a nutraceutical can be a good choice in these circumstances. A nanomedicine formulated by an engineered vaccine (to tackle virus) and nutraceuticals (to support postinfection via supporting immune systems) can be optimized as precision medicine.^28^ Further, this approach can be formulated according to patient health profiling, that is, personalized medicine. The time is now for an effective precision medicine approach for COVID‐19. This science has a formidable armamentarium going from AI, big data reservoirs, bioinformatic systems, and subtype sciences, leading to more specific therapies and thus better patient outcomes.

Health experts suggested that focus on multidisciplinary approaches is urgently required to properly manage Omicron variant‐based COVID‐19 pandemic, including a deeper understanding of Omicron‐SARS‐CoV‐2 infection, introducing AI to analyze bioinformatic to design new therapies or monitor the efficacy of off‐label drugs and diseases management strategies, increasing awareness about vaccination and booster program, biomedical/biotechnology‐based approaches focused on gene sequencing, developing efficient testing systems, and therapies of higher efficacy, and public–private partnership for making regulatory for socioeconomic balance and health wellness.

Suppose the pandemic from SARS‐CoV‐2 and its many variants is still ongoing despite vaccination coverage now exceeding 60% in almost all Western countries; in that case, it is necessary to (continue) investigate different approaches to immunization and treatment. The attention so far has been directed mainly to the epidemiology of the diseases and the vaccination, but the need to implement the attention to other disciplines to better understand virus evolution, the disease spread, course, and prognosis are necessary and will probably represent a game‐changing solution in the fight against the ongoing viral pandemic but also help respond to future infectious disease outbreaks.

## 4 CONCLUDING REMARKS AND FUTURE PERSPECTIVES

It has been investigated that Omicron infection is different than the COVID‐19 infection caused by other variants of SARS‐CoV‐2. Though it is less severe than Delta, it still has serious adverse effects on human health. Additionally, Omicron can reinfect the population who has COVID‐19 infection,^14^ or vaccinated people (both doses), and/or both the cases. These circumstances project Omicron infection as a serious concern, and its complete management needs special efforts and regulations, as summarized in Figure 3.

Although the Omicron variant may have the capacity to circumvent immunity from past infection, it is urgently required to explore how Omicron could avoid vaccine‐induced immunity and how it can lower immunity to infection on protection against severe illness and mortality. Regardless of efficacy, a full vaccination is recommended to minimize hospitalization and mortality rate, but how effectively antibodies elicited by existing vaccines can neutralize the Omicron variants is unknown and should be clearly investigated. Early availability of anti‐inflammatory, antivirals, and immunotherapies, such as MAbs, to manage Omicron infection is expected to lower hospitalization risk and mortality by 70% in high‐risk infected and exposed patients. As futuristic approaches, Pfizer and Moderna proposed a fourth booster dose (within 12 months of the third booster) possibly followed by other doses. This claim is supported by the health agencies in the direction of developing timely and appropriate therapeutics.

The emergence of the Omicron variant with its high‐level transmission and rapid spread worldwide, and its serious threat to many of the approved COVID‐19 vaccines and therapeutics, proved that the new variants of COVID‐19 are very likely to appear again, sooner or later. Therefore, this could be compelling enough for bio/pharmaceutical companies that there is an unmet need for the design and development of novel interventions and strategies that can predict the evolutionary trajectory of SARS‐CoV‐2 and its seriousness in case of the emergence of a deadlier coronavirus in the future. In both industrial and academic environments (preferably with tight junction and collaboration between both), much research must be done to prevent such infectious diseases in the future.

Dr. Christopher Murray, the director of Institute for Health Metrics and Evaluation, recently published a commentary in The Lancet^29^ pointing out that based on the analysis of global infections and the significant decrease in the level of severity, and considering the increase in the number of asymptomatic infections along with increasing the rate of vaccination and immunity worldwide, we can conclude that the end of COVID‐19 pandemic is near.^29^ Although this convinces a large number of governments and societies to slow down on the extraordinary testing, analysis, tracking, and measurements to control the transmission of COVID‐19, careful consideration should be taken into account globally to avoid the early emergence of a new recurrent pathogen, which possibly could be deadlier and more difficult to manage.

Some recommendations are focused on generating a big database based on large‐scale testing, sample collections, and gene sequencing. Such developed information process through AI will provide a better idea about the correlation of Omicron infection with race, gender, region, effectivity against available treatment, impact on the already sick or underlying health condition population, and support to authorities to develop effective planning. Besides, computational analysis of these data is also useful to investigate the possible mutations in the existing structure of Omicron, expected severity, novel targets needed to design new therapeutics, and assistance to pharma companies needed for developing new vaccines timely.

Besides prospects, in present scenarios, it is recommended that immunity be produced naturally, and vaccination may not be sufficient to protect us from Omicron infection. Therefore, the vaccination in support of booster doses (third and fourth doses, and its continuation if necessary) is the best recommendation to control this newly emerged phase of COVID‐19 infection. Last but not least, it is known in case of a pandemic that precaution and prevention are better than the cure, thus everyone must practice and participate in controlling this unprecedented situation, and the best way is to follow precautions, especially hygiene and masking.

## FUNDING INFORMATION

No direct funding is involved.

## CONFLICT OF INTERESTS

The authors declare no competing interests.

## AUTHOR CONTRIBUTIONS

All the authors contributed to articulating this article. E.M., A.K.D., L.T., and A.K. are involved in drafting the report. E.M. generated the Figures.

## ETHICS APPROVAL

Not applicable.

## Data Availability

Not applicable.
